# Acute Effect of Central Administration of Urotensin II on Baroreflex and Blood Pressure in Conscious Normotensive Rabbits

**DOI:** 10.3389/fphys.2017.00110

**Published:** 2017-02-23

**Authors:** Kyungjoon Lim, Yusuke Sata, Kristy L. Jackson, Sandra L. Burke, Geoffrey A. Head

**Affiliations:** ^1^Department of Neuropharmacology, Baker IDI Heart and Diabetes Research InstituteMelbourne, VIC, Australia; ^2^Department of Physiology, Monash UniversityClayton, VIC, Australia; ^3^Faculty of Medicine, Nursing and Health Science, Monash UniversityClayton, VIC, Australia; ^4^Department of Pharmacology, Monash UniversityClayton, VIC, Australia

**Keywords:** Urotensin II (SD1293), baroreflex, blood pressure, conscious rabbit, central effect

## Abstract

In the present study, we examined the effects of central administration of Urotensin II on blood pressure, heart rate, and baroreceptor heart rate reflexes in conscious normotensive rabbits. Preliminary operations were undertaken to implant a balloon cuff on the inferior vena cava for baroreflex assessments and to implant cannula into the lateral and fourth ventricle. After 2 weeks of recovery cumulative dose response curves to Urotensin II (10, 100 ng, 1, 10, and 100 μg) given into the ventricles, or Ringer's solution as a vehicle were performed on separate days. Injections were given each hour and baroreflex assessments were made 30 min after each administration. Analysis of the dose response curves to Urotensin II compared to vehicle administered into the lateral or fourth ventricle, indicated little change to blood pressure or heart rate. Analysis of the time course to the highest dose over a 30 min period revealed a small (−5 mmHg) depressor response maximal at 10 min when injected into the fourth ventricle but no effect when injected into the lateral ventricle. Baroreflex assessments made at each dose showed that there was no change in baroreflex sensitivity but that an increase in the upper plateau was observed when Urotensin was injected into the lateral ventricle and a tendency for a reduced lower heart rate plateau was observed after fourth ventricle administration. Clonidine administration in the fourth ventricle decreased blood pressure and heart rate, thus confirming catheter patency. In conclusion, our findings suggest that Urotensin II in the forebrain and brainstem may play a role in modulating cardiac sympathetic and vagal baroreflexes but only during large acute changes in blood pressure.

## Introduction

The cyclic dodecapeptide Urotensin II is a powerful agent to affect arteries (Ames et al., 1999; Maguire and Davenport, 2002). Urotensin II and its G-protein-coupled receptor, GPR14 are both expressed within the mammalian vasculature (Douglas and Ohlstein, 2000). It has been found that activation of GPR14 by Urotensin II results in a constriction of isolated blood vessels which is dependent on both species and anatomical origin, making Urotensin II an efficacious, sustained spasmogen of non-mammalian and mammalian isolated blood vessels (Nothacker et al., 1999; Douglas and Ohlstein, 2000; Douglas et al., 2000; MacLean et al., 2000; Maguire et al., 2000; Maguire and Davenport, 2002). Physiological role of Urotensin II includes induction of vasoconstriction, migration, and proliferation of vascular smooth muscle cell and control of cardiac output. Urotensin II is also linked in the progression of atherosclerosis, many types of cardiac disease, renal disease, and metabolic syndrome (Ross et al., 2010). In this regard, the cyclic structure of the peptide which is responsible for its biological action has been highly conserved from fish to mammals (Douglas and Ohlstein, 2000; Ross et al., 2010). *In vivo* studies show marked species differences in the actions of intravenously administered Urotensin II. In the rat, intravenous Urotensin II elicits a depressor response due to mesenteric and hindquarter vasodilatation associated with tachycardia (Gardiner et al., 2001; Lin et al., 2003). By contrast administration of Urotensin II to primates produces a powerful vasoconstriction (Ames et al., 1999) but blood pressure falls dramatically due to a cardiac depressor action. In humans, intravenous Urotensin II has no significant vasoconstrictor or vasodilator action on arteries or veins of small or medium caliber (Hillier et al., 2001).

There is now evidence to suggest that Urotensin II may play a role within the central nervous system (CNS) (Conlon et al., 1996; Chartrel et al., 1998). More specifically, GPR14 has been discovered in nuclei throughout the brain of many different species including rats (Dun et al., 2001), frogs (Coulouarn et al., 1998), and humans (Coulouarn et al., 1998). Immunohistochemistry has revealed that Urotensin II is found in the soma of cholinergic motoneurons (Dun et al., 2001). While some of the regions are involved in control of muscles, others such as the dorsal motor nucleus of the vagus and the nucleus ambiguous are known to be the source of cardiac vagal motor neurons and may play a role in regulating heart rate and therefore possibly cardiovascular reflexes. While studies so far suggest that Urotensin in the brain is likely to play a role in cardiovascular regulation, its precise role is not clear. In conscious rats pressor and tachycardic actions of Urotensin II have been reported (Lin et al., 2003) but in anesthetized rats, Urotensin II causes hypotension and bradycardia (Gibson et al., 1986). Direct microinjection of Urotensin II into the region of the brainstem of rats produced hypotension and bradycardia but no effect was observed when injected into the A2 region of the dorsomedial medulla (Lu et al., 2002). By contrast, microinjection into the forebrain nuclei such as the paraventricular or the arcuate nucleus evokes large increases in blood pressure and heart rate (Lu et al., 2002). Furthermore, studies in sheep show that ICV administration of small doses of human Urotensin II to sheep produces a long lasting increase in cardiac output, heart rate and blood pressure accompanied by increasing levels of stress and alerting hormones but little effect on vascular resistance (Watson et al., 2003). To date however there have been no reports of central administration of Urotensin II on cardiovascular reflexes. Furthermore, studies so far on its central actions have been limited to rats and sheep. Thus, the presence of the peptide and its receptors in pre-autonomic brain regions led us to hypothesize that the central administration of Urotensin II will affect the autonomic control of cardiovascular variables in the conscious rabbits. Hence, the aim of the current study was to investigate the effects of central Urotensin II on cardiovascular parameters and the baroreceptor heart rate reflexes in another species, namely conscious normotensive rabbits. The reflex is well-characterized in this species (Korner et al., 1972) being largely mediated by changes to cardiac vagus activity unlike the rat which has a larger sympathetic component (Head and McCarty, 1987). In addition, we examined both lateral ventricle and fourth ventricular administration to separate forebrain from hindbrain actions.

## Materials and methods

### Animals

Experiments were conducted in five naïve female rabbits and one male rabbit, bred from stock and housed at the Baker Heart and Diabetes Research Institute and whose weight ranged from 2.5 to 3.5 kg. Female rabbits are induced ovulators and as such do not undergo an estrous cycle (Adams and Ratto, 2013). In this regard, there are few if any differences in the cardiovasvular responses between sexes. The colony was derived from an original multi-colored English strain with “Dutch Belted.” Prior to surgery and post-operatively each animal was housed in individual standard rabbit cages, under conditions of constant ambient temperature, constant humidity and normal light/dark cycle (with the lights on from 0700 to 1900). Food and water were accessible *ad libitum* for the duration of the study. All procedures were approved by the Alfred Medical Research Education Precinct Animal Ethics Committee and conducted in accordance with the Australian Code of Practice for Scientific Use of Animals (Australian Code of Practice for the Care and Use of Animals for Scientific Purposes 7th Edition, 2004).

### Surgical preparation

All animals underwent three separate surgical operations performed under halothane Pharmachem, Australia) after induction with intravenous administration of propofol (10 mg/kg).

During the first procedure, a small perivascular silastic balloon was implanted around the intrathoracic inferior vena cava through a right thoracotamy while the rabbit was artificially ventilated. The exterior extremity of the tubing attached to the balloon was sutured under the skin between the scapulae. The lungs were reinflated, air removed from the chest cavity, the ribs drawn tightly together and the muscle and skin sutured.

Two weeks after the initial surgery, the six rabbits underwent a second surgical procedure. An intracerebroventricular catheter (Plastics One, Roanoke, VA, USA) was implanted into the lateral ventricle (coordinates from bregma; −3 mm lateral and −4 mm ventral) as described previously (Head and Williams, 1992).

Following the recovery period from the first two procedures, a fourth ventricular catheter was implanted as described previously (Head et al., 1983). Briefly, the atlanto-occipital membrane was exposed, and a small hole made with a 25G needle. A small vinyl catheter was inserted 8 mm through the hole allowing the tip to lie along the floor of the fourth ventricle. The end of the catheter was buried subcutaneously at the back of the rabbit's neck.

### Drugs

The drugs used were Human Urotensin II (Servier, Australia), L-phenylephrine HCl (Sigma Company, Australia), and clonidine HCl (Boehringer Ingelheim Pty Ltd, Australia). The doses of drugs were expressed in μg of the base. Drugs administered into the lateral ventricle or fourth ventricle were dissolved in Ringer's (Baxter, Old Toongabbie, NSW, Australia) solution while drugs given i.v. were dissolved in normal saline (Baxter, Old Toongabbie, NSW, Australia).

### Experimental procedures

Experiments were conducted in conscious rabbits held in a standard single rabbit holding box. Arterial pressure was measured from the central ear artery which was catheterized transcutaneously (Burke and Head, 2003).

Pulsatile arterial blood pressure was measured with a Statham P23ID strain gauge pressure transducer (Statham, Hato Rey, Puerto Rico) and HR was derived by a rate-meter triggered from the arterial pulse. Mean arterial pressure (MAP), HR and respiration rate were digitized and averaged over 2 s periods by computer using the LabVIEW programming language (National Instruments, Austin, TX, USA). Throughout the experiment, cardiovascular parameters were recorded on a six-channel polygraph (Grass Instruments, model 7D, Quincy, Massachusetts, USA).

### Experimental design

#### Protocol

The first experiment was conducted at least 14 days after implantation of the fourth ventricle catheter. Each rabbit underwent three experiments, each separated by 2 week recovery, during which a dose response curve to either Urotensin II into the lateral ventricle, Urotensin II into the fourth ventricle or a control experiment was performed which consisted of administering the same volumes of Ringer's solution (either via lateral or fourth ventricle). For each experiment, there was an initial 30 min acclimatization period and a 30 min period during which baseline baroreflex parameters were obtained in duplicate. Dose-response curves were performed using a range of five increasing doses of Urotensin II administered in 25 μL. The doses were 10 ng (7 pmol), 100 ng (72 pmol), 1 μg (0.7 nmol), 10 μg (7 nmol), and 100 μg (72 nmol) and thus covered 4 orders of magnitude. The dose range was based on a previous publication (Lin et al., 2003). Each dose was separated by a 60 min recording period during which a single ramp baroreflex assessment was made (between 30 and 45 min). The order of experiments was randomized and only one dose response curve was performed in the animal per experiment.

The HR baroreflexes were derived from slow ramp rises and falls in MAP by i.v. infusion of 0.5 mg/ml phenylephrine and inflation of the vena caval cuff respectively (Gaudet et al., 2000). At the conclusion of the third and final experiment for each animal, 5 μg/kg of clonidine was administered via the fourth ventricle to ensure the catheter was implanted correctly (Head et al., 1983).

### Data analysis

#### Hemodynamic data

Data was averaged over at least 10 min for the control periods, and 30 min following administration of the treatment.

#### Analysis of baroreflex curves

MAP and HR were averaged over 2 s intervals and fitted to a sigmoid logistic function to produce MAP-HR curves as described previously (Ricketts and Head, 1999). We used a non-linear regression program utilizing the Marquardt-Levenberg method to estimate a lower plateau which is minimum RSNA or HR, the HR range between upper plateau (which is a calculated maximum activation) and lower plateau, an upper and lower parameter defining the curvature and the median blood pressure or MAP at half the reflex range (BP50) (Ricketts and Head, 1999). The average range-dependent gain of the curve (G) was calculated HR range x curvature/4.

### Statistical analysis

Values were expressed as mean ± standard error of the mean (SEM) or mean difference ± standard error of the difference (SED). For all parameters, a repeated measure ANOVA was performed on the change (delta) from control values. The between treatment sums of squares was partitioned into two non-orthogonal contrasts comparing each treatment with vehicle. In addition, the between doses sums of squares was partitioned into a linear trend (indicating a dose response relationship) and the non-linear component. A Bonferroni adjustment of the t statistic was made to account for the multiple testing and lessen the likelihood of a type 1 error.

## Results

### Basal cardiovascular measurements

There were no differences in MAP or HR with Urotensin II injection into the lateral ventricle, into the fourth ventricle and control experiment over time (83.6 ± 4.1, 88.2 ± 5.4, and 81.6 ± 3.9 mmHg for MAP, 188 ± 5.3, 211.5 ± 14.6, and 207 ± 10.4 b/min for HR, respectively).

### Urotensin dose response curves

**Figure 1:**
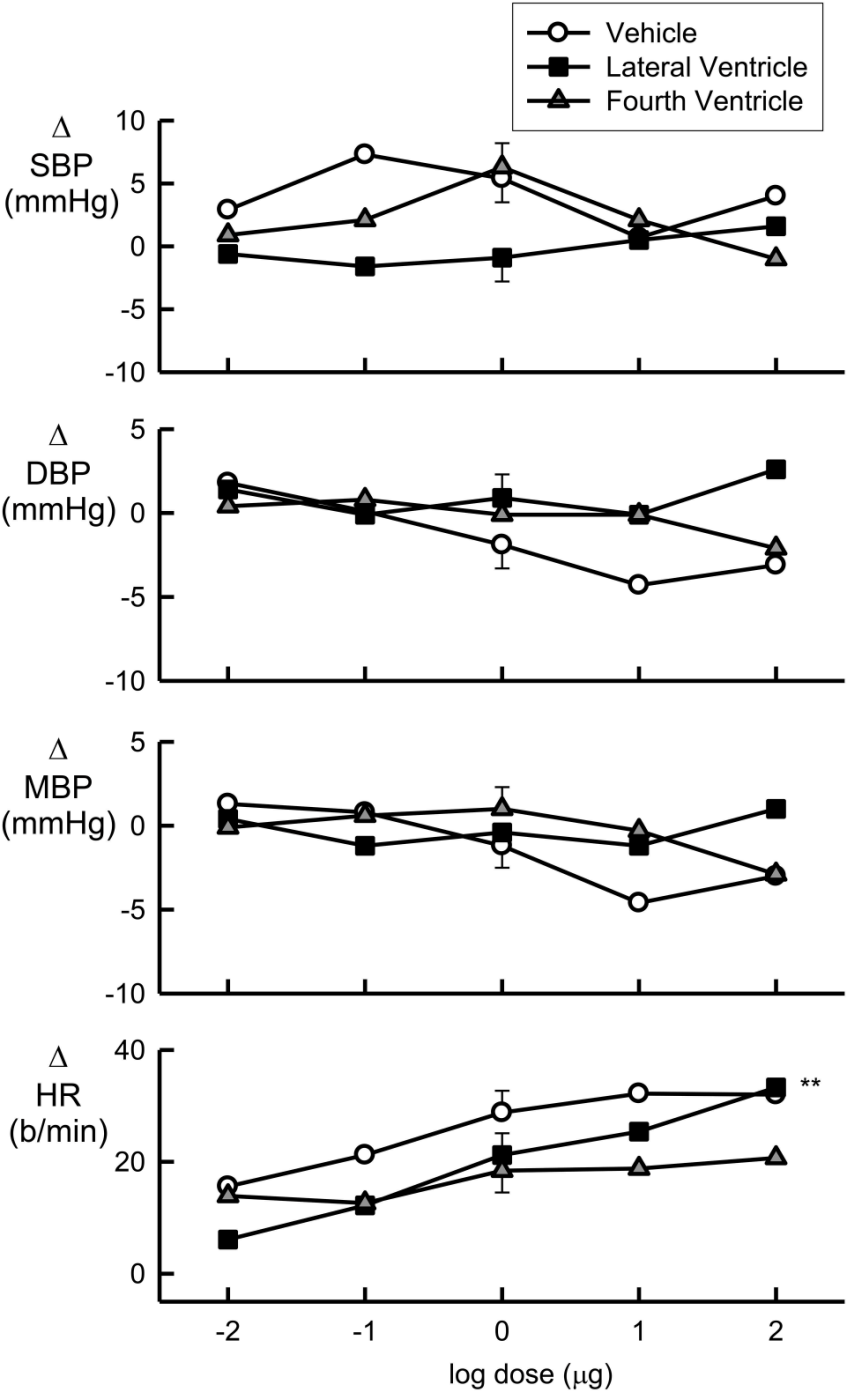
**Log dose response curves for Urotensin II and vehicle showing the changes in the baroreflex cardiovascular parameters of systolic blood pressure (SBP, mmHg), diastolic blood pressure (DBP, mmHg), mean blood pressure (MBP, mmHg), and heart rate (HR, beats per min), expressed relative to the initial control parameters**. Error bars are SEM indicating animal variance between doses within groups.

Increasing doses of Urotensin II administered into the fourth ventricle did not alter any of the cardiovascular parameters measured (Figure 1). While lateral ventricle administration of Urotensin II did not alter blood pressure, we did observe a dose dependent increase in HR (Figure 1, *F*_1, 70_ linear trend = 12, *P* < 0.01). The analysis of variance showed no difference in the effect of Urotensin on HR compared to vehicle administration (Figure 1, Table 1). This was because there was a tendency for HR to be elevated during the experiments compared to control for all treatments (average +26 b/min for vehicle, +20 b/min for lateral ventricle Urotensin and +17 b/min for fourth ventricle Urotensin).

**Table 1:** **Effects of treatment with Urotensin II administered in the fourth or lateral ventricles on cardiovascular parameters**.

	**Dose (μg)**						**Significance**	
	**0.01**	**0.1**	**1.0**	**10.0**	**100**	**SEM**	**Linear trend**	**Difference from Veh**
**VEHICLE**								
Systolic BP (mmHg)	2.9	7.3	5.4	0.7	4.0	1.9	NS	
Diastolic BP (mmHg)	1.8	0.1	−1.9	−4.3	−3.1	1.4	NS	
Mean BP (mmHg)	1.3	0.8	−1.2	−4.6	−3.0	1.3	NS	
HR (b/min)	15.6	21.2	28.8	32.2	32.0	3.9	NS	
**LATERAL VENTRICLE**								
Systolic BP (mmHg)	−0.6	−1.6	−0.9	0.5	1.6	1.9	NS	NS
Diastolic BP (mmHg)	1.4	−0.1	0.9	−0.1	2.6	1.4	NS	NS
Mean BP (mmHg)	0.4	−1.2	−0.4	−1.2	1.0	1.3	NS	NS
HR (b/min)	6.1	12.2	21.2	25.4	33.3	3.9	^**^	NS
**FOURTH VENTRICLE**								
Systolic BP (mmHg)	0.9	2.1	6.3	2.1	−1.0	1.9	NS	NS
Diastolic BP (mmHg)	0.4	0.8	−0.1	−0.1	−2.1	1.4	NS	NS
Mean BP (mmHg)	−0.1	0.6	1.0	−0.3	−2.9	1.3	NS	NS
HR (b/min)	13.9	12.6	18.4	18.8	20.7	3.9	NS	NS

Data expressed compared to control. Values are mean and SEM indicating within animal variance. Significance:

** *P < 0.01, NS, P > 0.05*.

### Time course of 100 μg urotensin II

**Figure 2:**
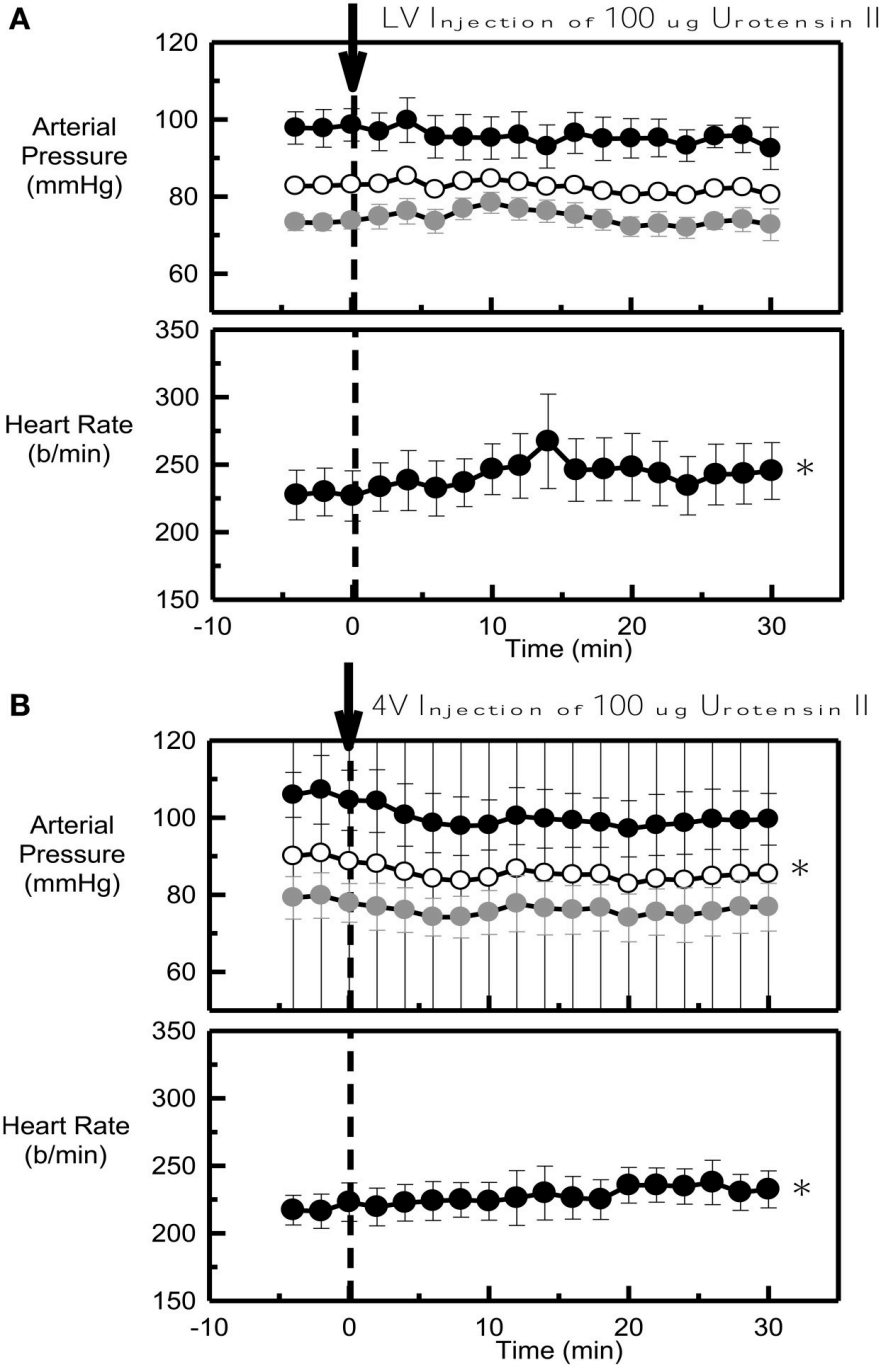
**Average systolic (black), mean (white) and diastolic (gray) arterial pressure (mmHg) and heart rate (b/min) before (control) and after 100 μg Urotensin II administered into the lateral ventricle (A)** and into the fourth ventricle **(B)** in six conscious rabbits (arrow at time 0). ^*^Indicates *P* < 0.05 compared to control. Error bars indicate the SEM between animals.

Urotensin II into the lateral ventricle (ΔMAP = −0.5 ± 0.8 mmHg) had no effect on blood pressure at any time within the 30 min period examined (Figure 2A). By contrast, this dose of Urotensin II administered into the fourth ventricle produced a small but clear hypotensive response (ΔMAP = −4.8 ± 1.0 mmHg, *P* < 0.001, Figure 2B) which reached a maximum at 10 min.

### Effect of urotensin of baroreceptor HR reflexes

**Figure 3:**
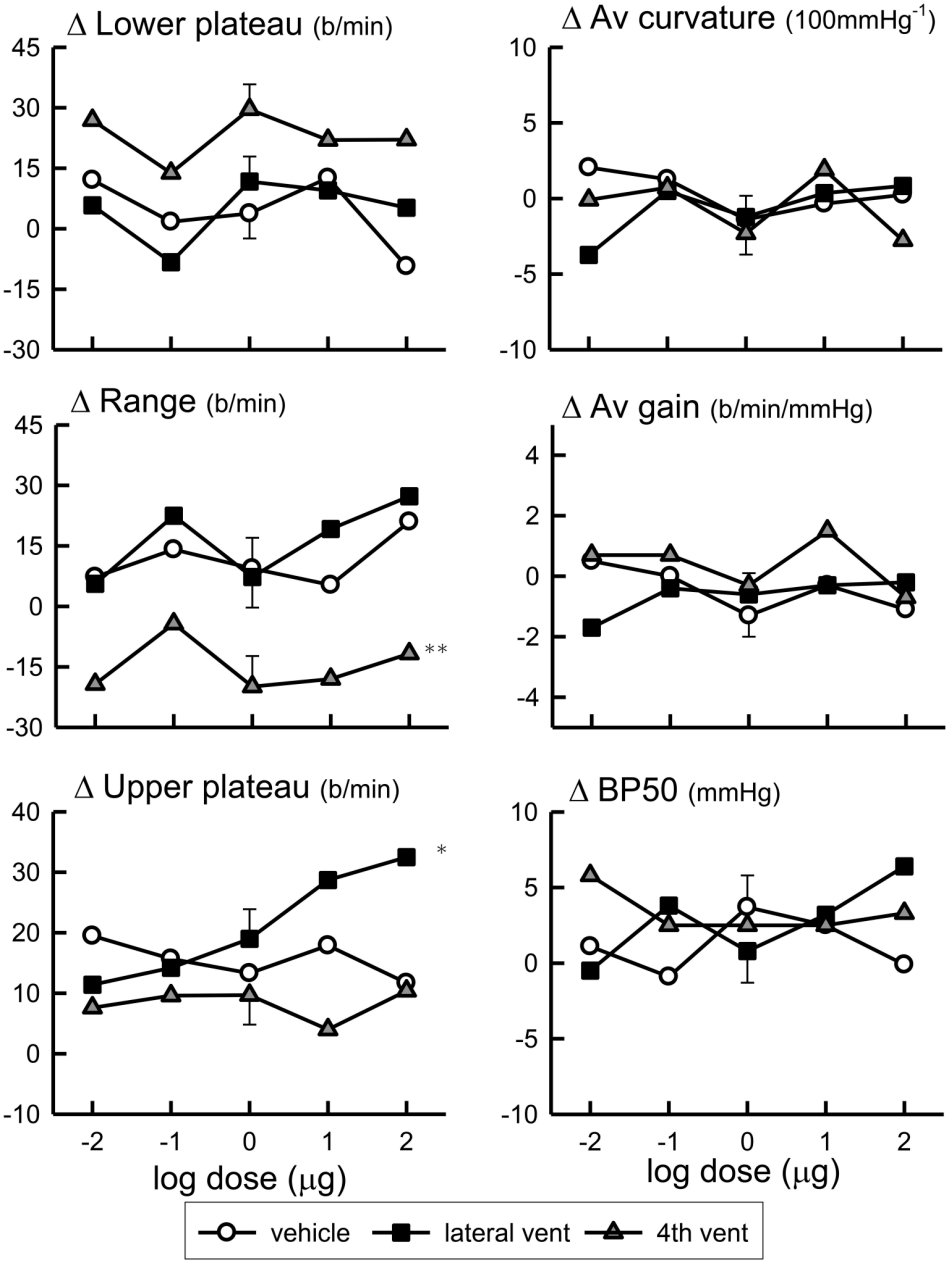
**Log dose response curves for Urotensin II and vehicle showing the changes in the baroreflex parameters of lower plateau (beats per minute), average curvature (100 mmHg^**−1**^), range (beats per min), average gain (beats per min per mmHg), upper plateau (beats per min), and BP50 (mmHg) expressed relative to the initial control parameters**. ^*^Indicates *P* < 0.05, ^**^P < 0.01.

Baroreflex assessments were made by using the rapid ramp method before and 30 min after each dose of Urotensin or vehicle. The blood pressure set-point (BP50) and the baroreflex sensitivity (average gain) did not change over the course of the experiment with repeated assessments during either the Urotensin II or vehicle administration protocols (Figure 3, Table 2). However, we did observe a lower HR range due to a change in the lower HR plateau in the animals given fourth ventricular Urotensin II (*P* < 0.01, Figure 3, Table 2). This effect occurred at all doses examined since there was no clear dose response relationship observed. Increasing doses of Urotensin II into the lateral ventricle increased the upper plateau of the baroreflex curve (*F*_1, 70_ linear trend = 5.3, *P* < 0.05, Figure 3).

**Table 2:** **Effects of treatment with Urotensin II administered in the fourth or lateral ventricles on baroreflex parameters**.

	**Dose (μg)**						**Significance**	
	**0.01**	**0.1**	**1.0**	**10.0**	**100**	**SEM**	**Linear trend**	**Difference from Veh**
**VEHICLE**								
Upper Plateau (b/min)	19.5	15.7	13.3	17.9	11.7	4.9	NS	
Range (b/min)	7.3	14.1	9.4	5.3	21.0	7.6	NS	
Lower Plateau (b/min)	12.1	1.7	3.8	12.6	−9.3	6.2	NS	
BP50 (mmHg)	1.1	−0.9	3.7	2.5	−0.1	2.1	NS	
Av. Gain (b/min/mmHg)	0.5	0.0	−1.3	−0.3	−1.1	0.7	NS	
Av. Curvature x 100 (mmHg ^−1^)	2.0	1.3	−1.4	−0.3	0.2	1.4	NS	
**LATERAL VENTRICLE**								
Upper Plateau (b/min)	11.4	14.2	19.0	28.7	32.5	4.9	^*^	NS
Range (b/min)	5.6	22.5	7.3	19.2	27.3	7.6	NS	NS
Lower Plateau (b/min)	5.8	−8.3	11.7	9.5	5.2	6.2	NS	NS
BP50 (mmHg)	−0.5	3.8	0.8	3.2	6.4	2.1	NS	NS
Av. Gain (b/min/mmHg)	−1.7	−0.4	−0.6	−0.3	−0.2	0.7	NS	NS
Av. Curvature x 100 (mmHg ^−1^)	−3.7	0.5	−1.2	0.4	0.8	1.4	NS	NS
**FOURTH VENTRICLE**								
Upper Plateau (b/min)	7.6	9.6	9.7	4.0	10.4	4.9	NS	NS
Range (b/min)	−19.3	−4.3	−19.9	−18.0	−11.7	7.6	NS	^***^
Lower Plateau (b/min)	26.9	13.8	29.6	22.0	22.1	6.2	NS	^**^
BP50 (mmHg)	5.8	2.5	2.5	2.5	3.3	2.1	NS	NS
Av. Gain (b/min/mmHg)	0.7	0.7	−0.3	1.5	−0.7	0.7	NS	NS
Av. Curvature x 100 (mmHg ^−1^)	−0.1	0.7	−2.3	1.9	−2.8	1.4	NS	NS

Data expressed compared to control. Values are mean and SEM indicating within animal variance. Significance:

* *P < 0.05*,

** *P < 0.01*,

*** *P < 0.001, NS, P > 0.05*.

**Figure 4:**
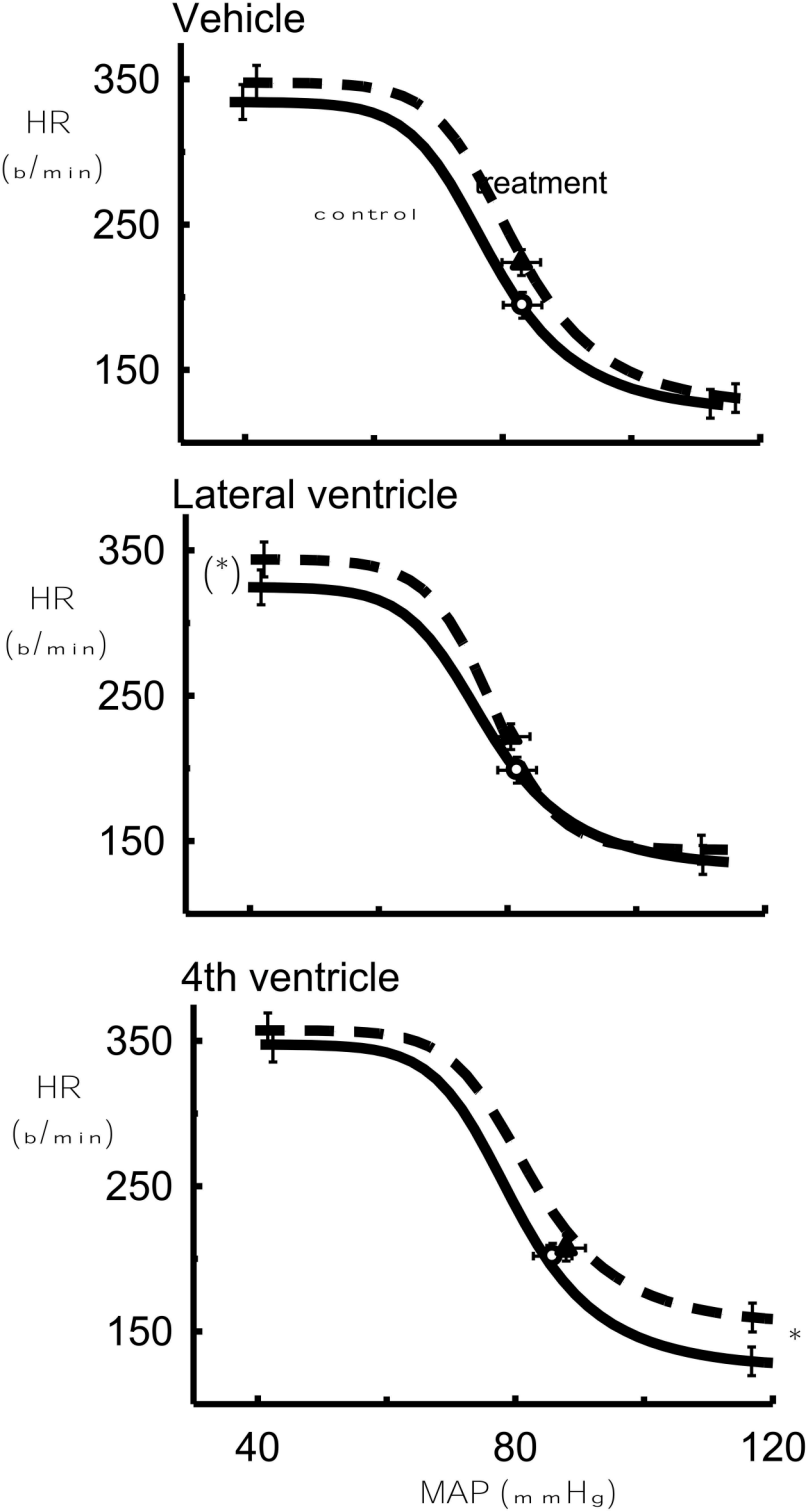
**The effect of 1 μg Urotensin II or vehicle in the fourth and lateral ventricles on the baroreflex curve averaged for 6 rabbits as compared to control**. ^*^Indicates *P* < 0.05.

**Figure 5:**
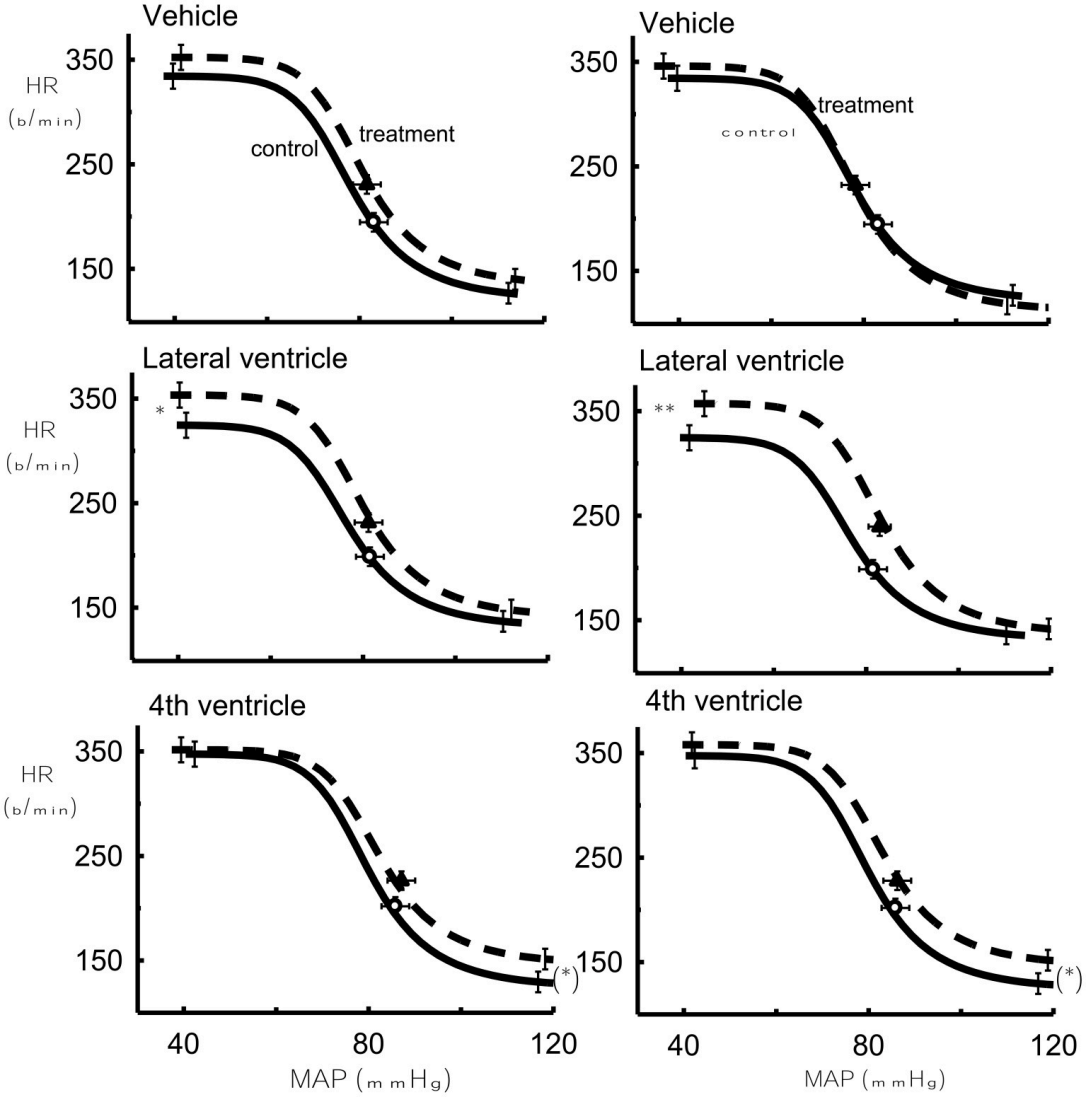
**The effect of 10 μg Urotensin II or vehicle (Left)** in the fourth and lateral ventricles and the effect of 100 μg Urotensin II or vehicle **(Right)** on the baroreflex curve averaged for 6 rabbits as compared to control. (^*^)Indicates *P* = 0.06, ^*^indicates *P* < 0.05, ^**^indicates *P* < 0.01.

The lower HR plateau was reduced after 1 μg Urotensin II into the fourth ventricle compared to the control (*P* < 0.05, Figure 4) whereas with 10 and 100 μg doses, there was a borderline significance when compared to the control (*P* = 0.07, Figure 5).

With the highest doses of Urotensin II given into the lateral ventricle (10 and 100 μg) we did observe there a significant increase in the upper HR plateau of the curve compared to the control curves (*P* < 0.05 and 0.01, respectively, Figure 5).

### Effect of clonidine

**Figure 6:**
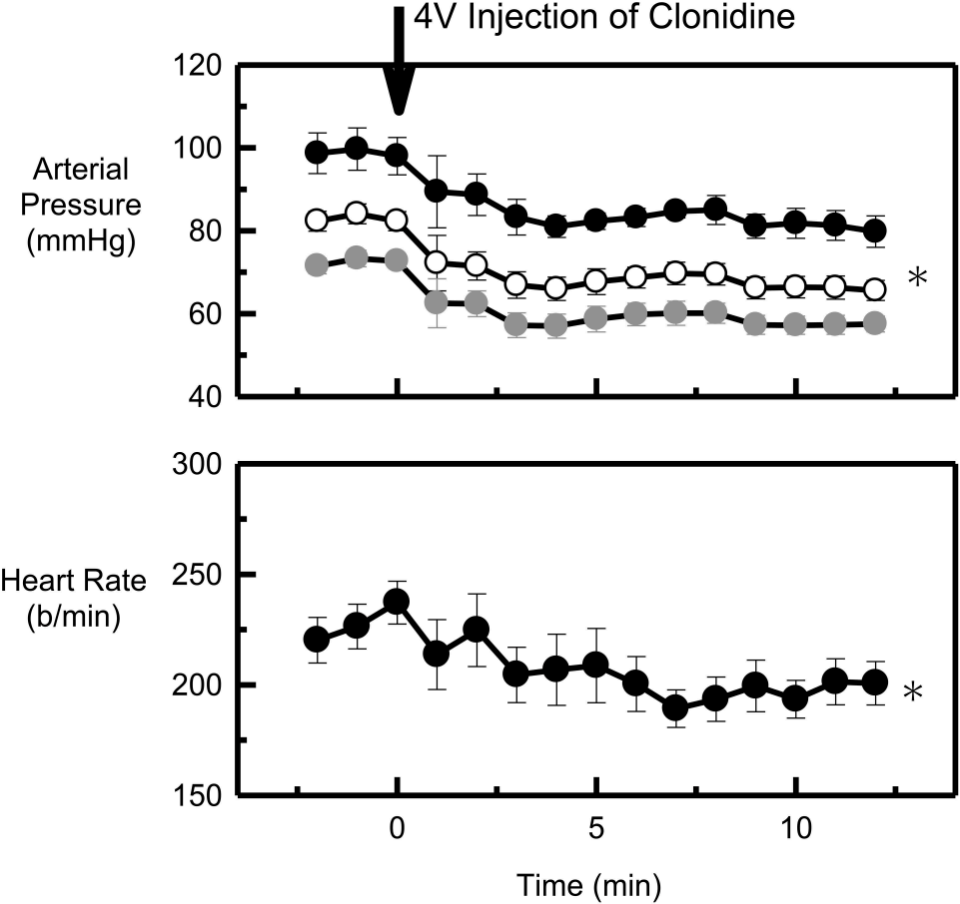
**Average systolic (black), mean (white) and diastolic (gray) arterial pressure (mmHg) and heart rate (b/min) before (control) and after 5 μg/kg clonidine administered into the fourth ventricle (arrow at time 0) of six conscious rabbits**. ^*^Indicates *P* < 0.05 compared to control. Error bars indicate the SEM between animals.

To confirm the patency of the fourth ventricular catheter clonidine was administered at the end of the last experiment in all 6 animals. Administration of 5 μg/kg of clonidine into the fourth ventricle elicited a rapid reduction in HR and blood pressure (Figure 6) in all animals tested.

## Discussion

The main findings of the present study were that Urotensin II administered into the fourth or lateral ventricle of conscious rabbits differentially affected the maximum and minimum plateaus of the baroreceptor HR reflex with few other changes observed to arterial pressure or HR. We observed a small depressor response to 100 μg Urotensin II administered into the fourth ventricle but the small increases in HR were not related to a specific effect of Urotensin II. The lateral ventricle administration increased the upper plateau of the HR curves which is where the sympathetic activity is maximally activated during hypotension. By contrast, the fourth ventricular administration inhibited the lower plateau which is predominately the vagal end of the curve. Taken together these results suggest that central administration of Urotensin II modifies autonomic control of heart rate but only when these reflex systems are responding close to maximal changes in blood pressure. Interestingly, the forebrain action of Urotensin II appears to facilitate the maximum cardiac sympathetic activity while the hindbrain action appears to be to inhibit the maximum cardiac vagal activity. The combined action would be to shift the curve upward.

To our knowledge, this is the first report of a central effect of Urotensin II on autonomic reflexes in conscious rabbits. It has been shown that autoradiographic labeling of brain slices indicate the presence of urotensin II binding sites in the lateral septum, bed nucleus of the stria terminalis, medial amygdaloid nucleus, anteroventral thalamus, anterior pretectal nucleus, pedunculopontine tegmental nucleus, pontine nuclei, geniculate nuclei, parabigeminal nucleus, dorsal endopiriform nucleus, and cerebellar cortex (Jégou et al., 2006). Importantly, Urotensin II immunoreactive cell bodies in the brainstem have shown dense labeling in the dorsal motor nucleus of the vagus and also the nucleus ambiguous among others (Dun et al., 2001). This would be a likely location for the actions of Urotensin II on the baroreflex as these are important areas involved in the vagal control of heart rate and suggests that Urotensin II as a possible neuromodulator through an autoreceptor, inhibits the cardiac vagus when they are most active. Clearly, the distribution of Urotensin II needs to be confirmed in the rabbit and electrophysiological studies would be necessary to confirm this hypothesis.

Previous studies examining the central cardiovascular actions of Urotensin II have been performed so far only with rats (Lin et al., 2003; Brailoiu et al., 2014), sheep (Watson et al., 2003), and conscious trout (Le Mevel et al., 1996; Vanegas et al., 2015). It has been reported that in rainbow trout (Oncorhynchus mykiss), central injection of Urotensin leads to aggression and anxiety-like behavior (Backstrom et al., 2011). In conscious rats, central administration of Urotensin II produces a short lasting (10–20 min) dose dependent pressor response and tachycardia (Lin et al., 2003) but in conscious sheep the tachycardia lasts for several hours (Watson et al., 2003; Hood et al., 2005). In the present study, we observed only a small depressor response in conscious rabbits but with a time course similar to that observed in conscious rats. The maximum dose we have used (100 μg = 72 nmol) is also similar on a per kg basis to those used in rats (10 nmol) (Lin et al., 2003) but considerably larger than that used in trout (0.5 nmol) (Le Mevel et al., 1996) and sheep (8 nmol) (Watson et al., 2003). At this stage, we cannot rule out that the small depressor response was due to a peripheral action of Urotensin II. However, we did not observe any effect of lateral ventricle administration of Urotensin II and while Urotensin II constricts isolated arteries in rabbits (Saetrum Opgaard et al., 2000) there have been no reports of systemic effects on blood pressure reported in this species. Urotensin II given systemically causes a biphasic response in sheep with an initial pressor response followed by a long lasting depressor response (Watson et al., 2003). By contrast in conscious rats Urotensin II produces a rapid depressor responses (Douglas and Ohlstein, 2000). In the rabbit, up to the very high doses we have used, there does not appear to be any evidence for a central pressor response to Urotensin II unlike other species. Indeed, the rabbit appears to be unique (so far) in showing some limited depressor activity in the hind brain.

In the present study we used the human form of Urotensin II but across phyla from fishes to primates there is a great deal of homology of the key cyclic region of the peptide which is responsible for its biological action (Coulouarn et al., 1998). It is unlikely that the relatively small changes we observed are due to incompatibility between the human and rabbit forms of the peptide since the human form has been used successfully to constrict rabbit arteries (Saetrum Opgaard et al., 2000). In conclusion, we suggest that Urotensin II in the forebrain and brainstem may play an important role in modulating cardiac sympathetic and vagal baroreflexes in rabbits.

## Author contributions

KL, YS, KJ, SB, and GH contributed to the analysis of the data and writing of the manuscript. KL and SB conducted animal experiments.

### Conflict of interest statement

The authors declare that the research was conducted in the absence of any commercial or financial relationships that could be construed as a potential conflict of interest.
